# MeCP2: A Critical Regulator of Chromatin in Neurodevelopment and Adult Brain Function

**DOI:** 10.3390/ijms20184577

**Published:** 2019-09-16

**Authors:** Kubra Gulmez Karaca, David V.C. Brito, Ana M.M. Oliveira

**Affiliations:** 1 Department of Neurobiology, Interdisciplinary Centre for Neurosciences (IZN), Heidelberg University, 69120 Heidelberg, Germany; guelmez@nbio.uni-heidelberg.de (K.G.K.);; 2 Donders Institute for Brain, Cognition and Behaviour, Radboud University Medical Center, 6525 EN Nijmegen, The Netherlands

**Keywords:** adulthood, chromatin, DNA methylation, epigenetics, MeCP2, neurodevelopment, Rett syndrome, transcriptomic profile

## Abstract

Methyl CpG binding protein 2 (MeCP2) was first identified as a nuclear protein with a transcriptional repressor role that recognizes DNA methylation marks. MeCP2 has a well-established function in neurodevelopment, as evidenced by the severe neurological impairments characteristic of the Rett syndrome (RTT) pathology and the MeCP2 duplication syndrome (MDS), caused by loss or gain of MeCP2 function, respectively. Research aimed at the underlying pathophysiological mechanisms of RTT and MDS has significantly advanced our understanding of MeCP2 functions in the nervous system. It has revealed, however, that MeCP2 has more varied and complex roles than previously thought. Here we review recent insights into the functions of MeCP2 in neurodevelopment and the less explored requirement for MeCP2 in adult brain function. We focus on the emerging view that MeCP2 is a global chromatin organizer. Finally, we discuss how the individual functions of MeCP2 in neurodevelopment and adulthood are linked to its role as a chromatin regulator.

## 1. Introduction

Methyl CpG binding protein 2 (MeCP2) is a member of a complex family of proteins, the methyl-CpG-binding domain (MBD) protein family, that bind to methylated cytosines. Among the MBD family, MeCP2 is the most abundant in the adult brain and establishes a link between DNA methylation and higher-order chromatin structure through interactions with chromatin modifiers [1]. MeCP2 has been extensively studied in the context of Rett syndrome (RTT) (OMIM identifier #312750), a severe neurodevelopmental disorder caused by mutations in the X-linked *MECP2* gene, that occurs approximately in 1 out of 10,000 live female births. This disorder is characterized by developmental regression, including arrested cognitive and motor development, and loss of acquired skills, such as language skills and purposeful hand use in affected females at around 6–18 months of age [2]. Patients also exhibit microcephaly, gait abnormalities, seizures, respiratory irregularities, hypotonia, and autonomic dysfunctions. In some cases, autistic-like behavior, such as social withdrawal and avoidance of eye contact, also appears at this stage; however, these symptoms often disappear and individuals become more social with age, except for those patients diagnosed with an autism spectrum disorder (ASD) associated with RTT [3]. Therefore, despite the until-recently accepted view of RTT as an ASD, RTT has not been listed as an ASD in the Diagnostic and Statistical Manual of Mental Disorders since 2013 [3]. In the case of RTT males, dysfunction of MeCP2 usually results in the death of the patient within the first 2 years of life. Interestingly, it has been reported that gain-of-function mutations in *MECP2* gene locus are also detrimental for life quality. Individuals that carry an extra copy of *MECP2* also suffer from a neurological disorder called MeCP2 duplication syndrome (MDS) (OMIM identifier #300260). Unlike RTT, MDS affects almost exclusively males since the duplicated copy can be silenced during X-chromosome inactivation in females. Similar to the RTT, the disorder is characterized by neurodevelopmental delay, mental retardation, and difficulties in motor and language skills [4]. Given that both upregulation and downregulation of the protein cause overlapping neurological dysfunctions, the levels of MeCP2 are strictly regulated during neurodevelopment and adulthood.

**Figure 1:**
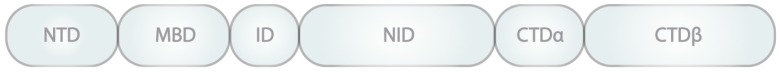
Schematic representation of the functional domains of methyl CpG binding protein 2 (MeCP2). MeCP2 contains an *N*-terminal domain (NTD), a methyl-binding domain (MBD), an intervening domain (ID), an NCoR-interacting domain (NID), and *C*-terminal domains α and β (CTDα and CTDβ, respectively).

MeCP2 is a nuclear protein characterized by several functionally annotated domains (Figure 1). These domains include a MBD and a NCoR-interacting domain (NID) that regulate the binding of MeCP2 to methylated DNA and the consequent transcriptional repression [5]. Furthermore, the protein contains a nuclear localization signal (NLS) that was thought to ensure the nuclear location of MeCP2. Interestingly, a recent report showed that the nuclear localization of MeCP2 may be determined not only by its NLS sequence, but also by the MBD domain [6]. Moreover, MeCP2 also contains an *N*-terminal domain (NTD), an intervening domain (ID), and the *C*-terminal portion that contains two DNA binding regions (CTD α and β). The latter are involved in the interaction with proteins that regulate chromatin structure [5,7].

MeCP2 was first described as a classical transcriptional repressor. However, several lines of evidence now indicate that MeCP2 exerts a transcriptional regulatory role through a more complex mechanism that involves global binding to DNA and regulation of the 3D-genomic and epigenomic landscape. Through the regulation of the neurons’ transcriptional profile, MeCP2 is thought to define structural and functional properties of the neurons both during neurodevelopment and in adulthood. In this review we summarize the current understanding of MeCP2 functions during neurodevelopment and in the adult brain with a focus on its role as a chromatin regulator. In the first part of the review, we address the functions of MeCP2 in neuronal development through the discussion of the neurological phenotypes present in RTT mouse models and RTT patient-derived neurons. In the second part, we review the less explored role of MeCP2 in the adult brain and argue that MeCP2 functions as a chromatin maintenance factor in mature neurons. We describe chromatin dysregulations in the absence of MeCP2 during adulthood and present studies that reveal a critical role for chromatin organization in neuronal function.

## 2. MeCP2 Function in Neurodevelopment

**Figure 2:**
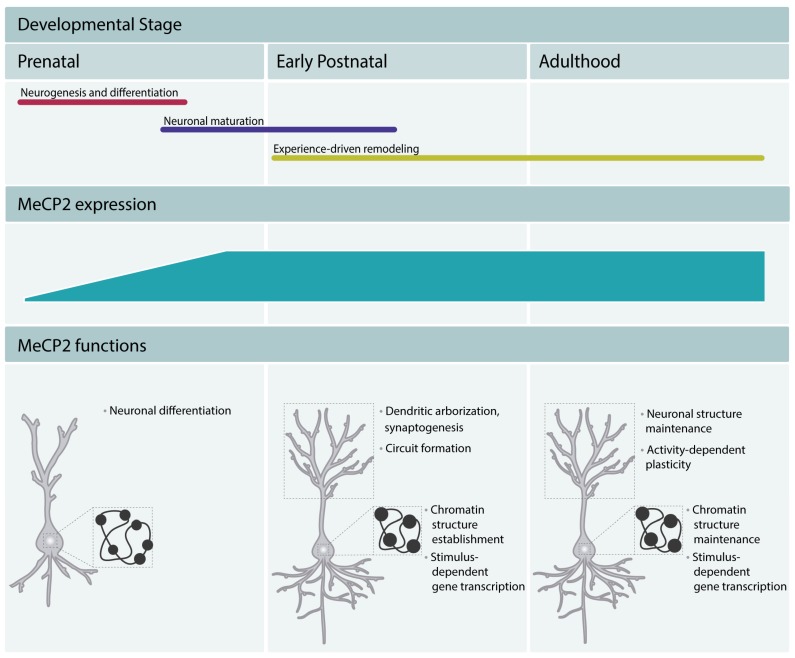
MeCP2 regulates brain development and maintains the function of mature neurons throughout adulthood. MeCP2 regulates neuronal differentiation in early embryonic development, neuronal maturation, and circuit formation. MeCP2 promotes chromocenter clustering during differentiation and maturation, and thus participates in the establishment of the typical chromatin structure of mature neurons (mature neurons present fewer and denser chromocenters, represented in the Figure as black dots in the nucleus). In adulthood, MeCP2 is a critical factor in the maintenance of the neuronal function. It maintains the chromatin structure and regulates the neuronal transcriptomic profile. Moreover, it appears to maintain a permissive state for stimulus-dependent gene transcription and regulate cognitive function.

Studies of MeCP2 expression pattern showed that it was present at higher levels in lung, spleen, and brain, with the highest levels in the latter. In mice, the levels of protein are low in the early stages of embryonic development [8,9], increase during the later developmental phases, and reach higher levels in mature neurons [9]. The levels of MeCP2 remain high throughout adulthood, which underscores its important function in the mature brain. In humans, MeCP2 expression starts in mid gestation and continues increasing until 10 years of age [9]. The increase in MeCP2 expression occurs after the neurogenesis and differentiation are completed and coincides with the stages of neuronal maturation, such as dendritic growth and branching and dendritic spine morphogenesis [10,11]. These early studies suggested a role for MeCP2 in the late stages of neurodevelopment and a function in mature neurons and implicated MeCP2 function as dispensable during early embryonic stages. The delayed expression pattern of MeCP2 was believed to underlie the apparently normal early development of RTT patients. The analysis of MeCP2 mouse knockouts [12,13] and mice with a truncating mutation in the *Mecp2* gene [14] further supported this view. The mutant mice appeared normal until 5 to 6 weeks after birth, after which phenotypes associated with neuronal dysfunction started to appear. However, more recent work demonstrated that MeCP2 expression can be detected in immature neurons of the prenatal cortex in mouse [8] and human induced pluripotent stem cell (iPSC)-derived neuronal progenitor cells (NPCs) [15]. Moreover, clinical studies revealed neurological alterations in RTT patients within the first months of life. Careful analysis of RTT patients showed altered general movements [16,17] and atypical speech-language capacities [18]. Hence, the expression of MeCP2 from the early stages of development through adulthood in both mice and humans and the presence of neurological deficits in RTT patients from early life suggest now that MeCP2 regulates all stages of neurodevelopment and adult brain function (Figure 2).

### 2.1. MeCP2 Regulates Neurogenesis and Differentiation

The expression of MeCP2 during early embryonic development suggests a role for the protein in neurogenesis and differentiation. Indeed, a few studies indicate that MeCP2 is required for these processes. Ectopic expression of MeCP2 in NPCs promotes neuronal differentiation while suppressing astrocytic fate determination [19]. In agreement with this study, neural stem cells derived from RTT patients differentiated preferentially into astrocytes. This phenotype was associated with altered expression of the astrocytic marker glial fibrillary acidic protein (GFAP) that resulted from the absence of MeCP2 binding to the *Gfap* gene. This suggests that MeCP2 regulates neurogenesis through the control of expression of cell fate genes [20]. Accordingly, reduced expression of neural markers was observed in differentiating mesenchymal stem cells obtained from an RTT patient [21]. Alterations in the differentiation of MeCP2-null embryonic neurons were also identified in mice [22]. Cobolli Gigli and colleagues (2018) used cortical NPCs generated from embryonic cortex of MeCP2 knockout (KO) mice and found that the expression of progenitor markers is more persistent in MeCP2-null cells, in line with permanence in an undifferentiated state [22]. Recently, Mellios and colleagues (2018) uncovered a mechanism that could underlie the effects of MeCP2 deficiency in early human neurogenesis and neuronal differentiation [23]. The authors used human RTT patient- and MeCP2 knockdown-derived monolayer and 3D neuronal culture models and found that the micro RNAs (miRNAs) miR-199 and miR-214 were significantly upregulated during early neurogenesis. MeCP2-deficient cultures exhibited deficits in dendritic complexity as well as reductions in the expression of neuronal markers. Moreover, increases in cellular proliferation were observed. These phenotypes were consistent with impaired proliferation and differentiation in MeCP2-deficient human cultured cells. The authors further showed that MeCP2 knockdown in the mouse embryonic brain elicited similar phenotypes. Interestingly, inhibiting miR-199 and miR-214 in RTT patient-derived neural progenitors improved the observed alterations in neuronal differentiation. Conversely, the overexpression of miR-199 or miR-214 in wild-type embryonic brains disturbed neurogenesis and neuronal migration. Taken together, this study provides compelling evidence that one mechanism through which MeCP2 regulates neurogenesis in mouse and humans is via the regulation of expression of miRNAs.

### 2.2. MeCP2 Regulates Synapse and Neuronal Circuit Formation

Neuronal maturation occurs in the later stages of neurodevelopment and comprises dendritic growth, spine morphogenesis, and the formation of neuronal circuits. MeCP2 expression increases during this developmental stage [9,11] and several studies have implicated MeCP2 in the regulation of neuronal maturation. MeCP2 KO mice [12,13] showed reduced brain weight and cortical and hippocampal volume [24,25,26], as well as reduced spine density and immature spine morphology [26]. Wang and colleagues performed a detailed analysis of dendritic complexity in MeCP2 mutant mice [27]. Reduced dendritic complexity was found in loss-of-function mouse models. Both MeCP2-null mice and mice bearing a truncating mutation in the *Mecp2* gene revealed reduced dendritic complexity. These changes were broader and presented an earlier onset in null mice. In this study MeCP2 gain-of-function did not lead to changes in dendritic complexity, suggesting that MeCP2 dosage may regulate differently dendritic morphology. Another study that analyzed organotypic slices from MeCP2 KO or overexpressing mice showed reduced dendritic arbor complexity and spine density [28]. The different phenotypes caused by MeCP2 gain-of-function in these two studies may be attributed to the different level of overexpression (two-fold [27] versus five-fold [28], respectively). The density of neurons is increased in MeCP2 KO mice [24,28], suggesting that the reduction in volume was due to reduced complexity and size of neurons rather than neuronal death.

The growth of dendrites and spines is fundamental to the development of neuronal circuits. Therefore, a likely consequence of the disruption of MeCP2 expression is impaired neuronal connectivity and synaptic transmission. Several studies have revealed neuronal circuit alterations brought on by the loss of MeCP2. In MeCP2 mutant mice the balance in excitatory/inhibitory (E/I) neurotransmission is disrupted. Using whole-cell patch-clamp recordings in cortical slices, Dani et al. (2005) showed that spontaneous activity of pyramidal neurons is reduced in MeCP2-mutant mice [29]. They found that the cortical E/I balance is shifted to favor inhibition over excitation. In contrast, Calfa and colleagues (2015) described impaired synaptic inhibition and E/I balance in area CA3 of acute slices from symptomatic MeCP2 KO mice [30]. This was associated with reduced expression of GABAA receptors and increased expression of GluA1 subunits in the CA3 of mutant mice. These changes may underlie the neuronal dysfunctions observed in RTT patients and mouse models. For example, a hyperactive hippocampal network may contribute to the limbic seizures observed in MeCP2 mouse mutants and RTT patients, whereas reduced excitation in cortical motor regions may lead to motor dysfunction. Belichenko and colleagues (2009) reported that the morphological alterations in MeCP2 mutant mice differ from region to region, which could possibly account for the region-specific changes in the function of neural circuits. The increase of expression of MeCP2 during neuronal maturation together with the onset of most neurological symptoms in RTT patients and mouse models at this stage strongly supports a critical function for MeCP2 during this phase. However, given the recent evidence that MeCP2 affects the early stages of neurodevelopment, the contribution of neuronal deficits that start during embryogenesis to the observed phenotypes in mature neurons should be considered.

### 2.3. MeCP2 Fine-Tunes Gene Expression and Establishes the Neurons’ Chromatin Landscape

To uncover the cellular mechanisms by which MeCP2 regulates neuronal development and function, several studies focused on its role as a transcription regulator and attempted to identify target genes. Surprisingly, microarray analysis of brain tissue from MeCP2 mutant mice obtained during symptomatic and pre-symptomatic phases revealed only very subtle expression changes [8,31,32,33,34]. These findings led to the speculation that small changes in many mRNAs may underlie the observed phenotypes. Differentially expressed genes were predominantly upregulated [32], consistent with a transcription repressor function for MeCP2. Furthermore, Urdinguio and colleagues (2008) found that most upregulated genes were bound by MeCP2, thus suggesting a direct regulation [34]. The discovery of only subtle changes in gene expression could be explained by a masking effect as a result of whole brain tissue analysis. Indeed, other studies that analyzed gene expression in selected brain regions of MeCP2 mutant mice, such as the hypothalamus or cerebellum, identified several genes with dysregulated expression [28,35]. The authors found that the majority of dysregulated genes were downregulated in MeCP2 KO mice, or upregulated in MeCP2-overexpressing mice. The positive correlation was in contrast to previous studies and suggested a transcription activator role for MeCP2 in the hypothalamus. This study identified an interaction between MeCP2 and the transcription factor cAMP response element binding protein (CREB) and proposed that MeCP2 may also function as a transcription activator through the interaction with transcription factors. In agreement with the previous studies the magnitude of the changes was small, suggesting a fine-tuning function. The authors further reported that the gene expression changes were in general smaller in MeCP2 KO mice compared to MeCP2-overexpressing mice, which may explain the low discovery rate obtained in previous studies using tissue from MeCP2 KO mice. A recent study performed transcriptomic analysis of MeCP2 mutant mice using an approach that allowed overcoming the cellular heterogeneity of the brain [36]. The authors used a Cre-LoxP-based recombination system that permitted transcriptional profiling of different cell types. This study revealed that MeCP2 regulates the expression of different genes in excitatory and inhibitory neurons. Moreover, the gene-expression profiles of RTT mouse models that contained point mutations (T158M, R106W) also revealed limited overlap between excitatory and inhibitory neurons, thus indicating that MeCP2 regulates a distinct genomic program in different cell types in both normal and RTT-associated conditions. Interestingly, the severity of the phenotype correlated with the number and degree of misregulated genes. This study demonstrated the complexity of MeCP2 function as a transcription regulator and underscored the need for analyzing different cell types independently in order to obtain a clear picture of MeCP2 target genes. It further suggests that a contributing factor to the low discovery rate in previous studies may be the use of whole tissue.

The abovementioned studies performed gene expression analysis using postnatal tissue. It should be noted however that gene expression changes in MeCP2 KO mice are readily identified during embryonic development [8]. Bedogni and colleagues identified a small set of genes which expression was dysregulated as early as embryonic day 15. These genes coded for glutamatergic receptors and ionic channels which have established roles in neuronal physiology. Using cultured embryonic cortical neurons the authors demonstrated that MeCP2 depletion and consequent gene expression changes in this developmental stage were associated with impairments in neuronal function; MeCP2-null neurons displayed reduced nuclei and impaired responses to neuronal activity. The observed reduced calcium transients in response to electrical stimulation were likely a result of the impaired expression of receptors and channels that regulate stimulus-dependent calcium influx. Importantly, the authors further reported that the changes in expression of glutamatergic receptors and cationic channels were present in vivo in embryonic tissue. This study thus revealed a requirement for MeCP2 in gene expression regulation and neuronal function from the early stages of neurodevelopment.

Transcriptomic analysis of MeCP2 mutant tissue has consistently revealed subtle changes in gene expression. This is consistent with a fine tuning function in transcriptional regulation and questioned the view that MeCP2 acts as a classical transcriptional regulator. Indeed, more recent studies showed that MeCP2 regulates gene expression through a more complex mechanism. MeCP2 binds broadly across the neuronal genome and is nearly as abundant as the histone octamer [37,38,39]. The absence of MeCP2 leads to global changes in neuronal chromatin structure, including increased histone acetylation [14,37]. In light of these findings, it was speculated that MeCP2, rather than acting as a gene-specific transcription factor, functions as a global regulator of the chromatin structure that is required to reduce aberrant transcriptional events allowing the transcriptional machinery to function properly [37]. Further studies, including the analysis of animal models that mimic MeCP2 mutations present in RTT patients, supported the view that MeCP2 functions as a chromatin organizer and allowed for uncovering the responsible functional domains and interacting partners. Transgenic mice that bear a point mutation in amino acids 270 or 273 (R270X or G273X, respectively) revealed that the AT hook 2 domain of MeCP2, absent in R270X transgenic mice, regulates chromatin compaction [40]. In another study, Bird and colleagues identified the 302–306 amino acid stretch as a recruitment surface of the chromatin regulator NCoR/SMRT [41]. Moreover, MeCP2 has been reported to antagonize the binding of H1 histone linker protein to nucleosomes, and to promote tertiary chromatin structures, similar to H1 protein [42]. Furthermore, MeCP2 has been shown to promote the formation of chromatin loops through its interactions with the alpha-thalassemia/mental retardation syndrome X-linked protein (ATRX) and the CCCTC-binding factor (CTCF) (e.g., at maternal allele of H19 ICR) [43]. Additionally, Brero and colleagues (2005) showed that chromocenter formation correlates with myogenic differentiation and increased levels of MeCP2 in muscle progenitor cells [44]. The exogenous expression of MeCP2 caused a dose-dependent clustering of chromocenters, indicating causality between MeCP2 levels and chromatin rearrangement. In agreement with a function for MeCP2 in chromocenter clustering, two studies showed that MeCP2 is necessary for heterochromatin reorganization and chromocenter clustering also during neuronal differentiation [45,46]. Furthermore, Agarwal and colleagues (2011) performed a systematic study that characterized 21 RTT MeCP2 mutations in terms of their ability to cluster heterochromatin [47] and found that the majority of the mutations disrupt chromocenter clustering. Thus supporting the view that chromatin alterations are linked to RTT symptoms.

Taken together, these findings suggested that MeCP2 functions as a chromatin architect during neuronal differentiation with an important role in the establishment and maintenance of the neurons’ differentiated chromatin state. Therefore, an emerging view is that the transcriptional regulatory role of MeCP2 is likely connected to its function as a chromatin organizer [48]. By affecting the global chromatin structure, MeCP2 fine-tunes and maintains a delicate balance of neuronal gene expression. In the absence of MeCP2, subtle but widespread changes in expression of MeCP2-regulated genes possibly result in RTT symptoms.

Recently it was discovered that in the brain, in contrast to other somatic tissues, methylated cytosines do not only occur in a CpG context, but also in CpH (H=A/C/T) [49,50,51]. A large number of mCpH were identified in the adult mouse dentate gyrus (DG) [52] and frontal cortex [49] and it was shown that genes enriched for high mCpH were conserved between mice and humans [52]. mCpH in neurons was found to be established during postnatal development of hippocampal and cortical regions and to be maintained throughout adulthood [49,52]. This is in contrast to mCpG, that appears to be established during early development and remain constant across time. The analysis of human tissue revealed that, similar to mice, mCpH is present at high levels in adult as compared to fetal brain [49,52]. Intriguingly, the timing of establishment and maintenance of mCpH coincides with the MeCP2 expression increase, and indeed it was shown that MeCP2 binds mCpH [39,52]. Another study found that in the postnatal brain, MeCP2 regulates the expression of genes that are enriched for mCpH rather mCpG [39]. The binding of MeCP2 to mCpA appeared to be of higher affinity than to mCpC or mCpT. Furthermore, MeCP2 binding was enriched at gene bodies that have high levels of mCpA [53]. It was found that MeCP2 at these sites has a repressive role on the expression of these genes and that the genes regulated by MeCP2 are longer than the genome wide average. The authors observed an upregulation of these genes in the brain of MeCP2 knockout mice, a finding that was later confirmed by another study [54]. In agreement with earlier gene expression studies, the magnitude of expression changes between wild-type and knockout mice was small [53]. A follow-up study showed that the repressive effect of MeCP2 is proportional to the total number of methylated CpA within each gene and proposed that MeCP2 represses transcription of long genes by hindering transcriptional elongation [55]. It important to note that a recent study showed that long genes were downregulated when nuclear RNA was used for RNA sequencing analysis, but upregulated when whole-cell RNA was profiled [36]. This indicates that post-transcriptional changes may occur and highlights the need to include the analysis of nuclear RNA profiling when attempting to identify direct effects of MeCP2 in gene expression. Overall, several lines of evidence show that during the later stages of brain development, mCpH marks build up in a subgroup of genes, whereas mCpG remains relatively unchanged. As MeCP2 accumulates during neuronal maturation, substantial amounts of MeCP2 occupy those mCpH sites to influence transcription. The writing of mCpH marks and subsequent recruitment of MeCP2 during postnatal development may constitute another layer of transcriptional control that underlies neuronal maturation and the maintenance of a mature neuronal state throughout adulthood.

### 2.4. MeCP2 Regulates Experience-Dependent Gene Expression during Postnatal Neuronal Development

It is well established that environmental stimulation is critical to shape and refine neuronal circuits during early childhood [56]. Therefore, another means through which MeCP2 may control synaptic development and maturation during the postnatal stage may be through the regulation of stimulus-dependent gene transcription. Two seminal studies showed that the activity-dependent expression of brain-derived neurotrophic factor (Bdnf) is regulated by MeCP2 [57,58]. It was shown that in primary cortical cultures MeCP2 is bound to the *Bdnf* promoter repressing its expression. Upon neuronal activity, in a phosphorylation dependent manner, MeCP2 is released from the *Bdnf* promoter facilitating transcription [57]. The release of MeCP2 binding to *Bdnf* promoter was associated with a decrease in CpG methylation in this gene locus [58]. A following study identified a specific aminoacid residue (S421) in MeCP2 that is phosphorylated in response to neuronal activity [28]. The authors showed that this phosphorylation is required for neuronal activity-triggered Bdnf expression. Moreover, they showed that the phosphorylation of MeCP2 at S421 is required for MeCP2-dependent regulation of dendritic and spine morphogenesis. Thus, suggesting that MeCP2 is a critical player in the maturation of neuronal connectivity guided by neuronal activity. These and other studies have identified *Bdnf* as a MeCP2 target gene. Bdnf is a secreted protein that promotes many aspects of experience-dependent synaptic development [59]. This suggests that *Bdnf* may be one effector gene through which MeCP2 regulates neurodevelopment and maturation. Supporting this idea is a study showing that Bdnf mutant mice show phenotypic similarities to MeCP2 mutant mice; Bdnf mutant mice exhibit smaller brain weight and neuronal size accompanied by motor deficits. The expression of Bdnf in MeCP2 mutant mice partly rescued the reduced brain size, delayed the onset of neurological deficits such as motor impairments, and led to significant increases in lifespan [60].

To further investigate the requirement of MeCP2 S421 phosphorylation for nervous system development, Cohen and colleagues (2011) generated a knockin mouse line in which S421 is converted to alanine to prevent phosphorylation of this site [38]. The characterization of these mice revealed increased complexity of the distal apical dendrites of cortical layer V and a shift in E/I balance in favor of inhibition in cortical circuits, thus indicating that MeCP2 phosphorylation is required for proper cortical dendritic patterning and circuit formation. The authors assessed the requirement of the phosphorylation to DNA binding and expression of target genes. Surprisingly, they observed that the MeCP2 DNA occupancy was similar in unstimulated and membrane depolarized neurons and that in both conditions MeCP2 binds broadly to the neuronal genome. This confirmed other studies that have also found broad distribution of MeCP2 [37], but appeared to contradict the previous view that upon neuronal activity, MeCP2 is phosphorylated and released from the regulatory regions of target genes such as *Bdnf* [57,58]. Moreover, microarray analysis of cortical neurons obtained from wild-type or MeCP2 S421A mice showed no significant differences in the levels or kinetics of gene induction in response to neuronal activity. The authors suggested that MeCP2 S421 phosphorylation in response to neuronal activity, facilitates a global chromatin response rather than serving as a gene-specific transcription regulatory mechanism. Therefore, the disrupted dendritic development and circuit formation in the MeCP2 S421A mice did not appear to be due to altered gene expression; hence, the precise mechanism underlying these deficits remained to be understood.

Other activity-dependent phosphorylation sites in MeCP2 have been identified, namely S86, S274, and T308 [61]. Different extracellular stimuli differentially induce phosphorylation at these sites suggesting that differential phosphorylation of MeCP2 could mediate the response to various stimuli. MeCP2 T308 phosphorylation disrupts the interaction with the NCoR repressor complex, thereby reducing MeCP2-mediated transcription repression. This phosphorylation did not appear to alter MeCP2 binding to the DNA, but rather the recruitment of chromatin regulators. MeCP2 T308A knockin mice have altered activity-dependent expression of plasticity-related genes and neurological deficits characteristic of RTT syndrome, including reduced brain weight, reduced motor function, and reduced seizure threshold. Although MeCP2 phosphorylation is the most characterized, the protein was also found to be SUMOylated [62], glycosylated [63], acetylated [64,65], and ubiquitinylated [64] (for more details see [66]). Taken together, these studies suggest that one mechanism through which environmental stimuli shapes neurodevelopment is via MeCP2 posttranslational modifications that determine the genomic recruitment of chromatin regulators and lead to regulation of chromatin architecture and of gene expression.

## 3. MeCP2 Function in the Adult Brain

### 3.1. MeCP2 Is Required for Adult Brain Function

In contrast to neuronal development, MeCP2 functions during adulthood are less understood. The majority of the studies sought to mimic RTT phenotypes in rodent models, thus performed germline deletion or manipulation of MeCP2. Although this proved extremely helpful to gain insights into the mechanisms underlying the pathophysiology of RTT, these experimental models did not allow the dissection of MeCP2 functions in the adult brain as the observed phenotype was confounded by neurodevelopmental abnormalities. A few studies have started to explore the MeCP2 functions in the mature brain by developing experimental models that restricted MeCP2 manipulations to the adult stage. Early studies performed by Nelson and colleagues (2006) revealed that elimination of MeCP2 through a lentiviral approach in cultured mature hippocampal neurons impaired synaptic plasticity in these neurons [67]. Later on, it was shown that post-birth re-expression of MeCP2 in MeCP2 KO mice decelerated RTT-like phenotypes in these mice [68] and re-activation of MeCP2 expression in MeCP2 KO mice reversed the already advanced dysfunctions in mature neurons [69]. These studies provided the first hints for the involvement of MeCP2 in the adult brain function. To explore this further, McGraw and colleagues (2011) generated a conditional KO mouse line in which MeCP2 is selectively deleted in adult mice (from postnatal day 60). The authors found that adult MeCP2 KO mice exhibit hypoactivity, motor and gait abnormalities, and impaired nesting behavior, as well as disrupted learning and memory abilities, similar to models in which MeCP2 was depleted early on [70]. These findings demonstrated that MeCP2 is required for proper functioning of the brain in adulthood and that the symptoms observed in RTT models are not solely due to loss of MeCP2 function during infancy.

Cheval and colleagues (2012) investigated whether MeCP2 is equally required throughout adulthood, or whether there are vulnerable phases for MeCP2 depletion [71]. For that, the authors depleted MeCP2 from mouse brain at 3, 11, and 20 weeks after birth and showed that two age windows, 8–14 weeks and 30–45 weeks, are particularly sensitive to the presence of MeCP2 in the brain [71]. They found that regardless of the age of MeCP2 depletion, mice showed RTT-like phenotypes and premature death; however, the onset of the symptoms was earlier when MeCP2 was inactivated at the ages of 11 or 20 weeks [71]. Using a similar approach, Nguyen, Du and colleagues (2012) showed that deletion of MeCP2 from the brain of 5-week or 10-week-old mice caused RTT-like symptoms both in female and male mice [24]. Interestingly, the kinetics for the onset of the symptoms did not differ when MeCP2 was depleted at late juvenile or adult state, suggesting an absolute requirement for MeCP2 for brain maturation and maintenance [24]. In addition to the previously identified critical windows for MeCP2 requirement in adult brain, Du and colleagues (2016) identified another developmental stage, transition from adolescent to adult stage (at around 15 weeks after birth), that critically requires MeCP2 [72]. MeCP2 loss at this stage caused severe RTT-like symptoms and lethality within few days [72], which is in contrast to previous studies reporting median survival time of 13–39 weeks [24,70,71]. The discrepancy between these studies regarding the survival rates and the kinetics of the symptoms could be dependent on the degree (i.e., rate and onset) of MeCP2 depletion from the brain, likely due to the mouse model used in the study, or differential application of tamoxifen. Collectively, these studies provided evidence that adult MeCP2 is indispensable for proper functioning and maintenance of the brain. However, MeCP2 was deleted from the whole organism or brain, and MeCP2 adult KO mice showed severe motor and breathing abnormalities that were incompatible with life. Due to these phenotypical limitations, the role of MeCP2 in specific behavioral tasks during adult life could not be addressed.

To investigate the MeCP2 requirement in specific brain functions, regional- and temporal-specific manipulation of MeCP2 was performed. For instance, to investigate the role of MeCP2 in learning and memory processes in the adulthood, Gulmez Karaca and colleagues (2018) used RNA interference to reduce the levels of MeCP2 specifically in the dorsal hippocampus of young-adult mice (8 weeks of age) and performed a detailed analysis of the mice behavioral performance [73]. The authors found that MeCP2 is selectively required for long-term but not short-term memory processes in adult mice and that hippocampal MeCP2 knockdown specifically impairs some forms of memory (such as object-place recognition and trace-fear conditioning), leaving highly salient memory forms intact (such as contextual-fear conditioning) [73]. Interestingly, Swiech and colleagues (2015) used the CRISPR-Cas9 technology to edit the MeCP2 locus and induce MeCP2 knockdown in the DG of the hippocampus in the adult, and showed that MeCP2 in mature DG is critical for contextual-fear conditioning [74]. Importantly, both studies confirmed that disruption of MeCP2 specifically in mature hippocampus did not alter anxiety-like behavior or locomotion abilities in mice, allowing a clear assessment of hippocampus-dependent memory performance [73,74]. The discrepancy in the findings concerning the requirement of MeCP2 for contextual-fear conditioning is present in several studies [75,76,77,78]. It likely reflects differences on the experimental protocol used in the different studies.

Deng and colleagues (2010) showed that a critical function for MeCP2 in the adult brain is not restricted to the hippocampus. Bidirectional manipulation of the levels of MeCP2 by lentiviral expression in the nucleus accumbens (NAc) of adult mice and revealed that MeCP2 in mature NAc regulates psychostimulant-triggered locomotion and its rewarding properties [79].

Several studies showed that MeCP2 removal from whole brain at the age of 5, 10, or 15 weeks resulted in shrinkage of the brain and an impaired dendritic arborization and spine density in hippocampal pyramidal neurons [24,72]. Intriguingly, knockdown of MeCP2 in the hippocampus at the age of 8 weeks did not alter the dendritic complexity or spine density of mature CA1 pyramidal neurons [73]. These findings suggest that partial loss of MeCP2 does not primarily alter dendritic morphology during adulthood and imply that the observed synaptic plasticity impairments in MeCP2 disruption may be a result of nuclear abnormalities. This view is further supported by decreased neuronal nuclei sizes observed in mature CA1 neurons depleted of MeCP2 expression [72]. Furthermore, acute reduction of MeCP2 levels was sufficient to disrupt the developmentally-established chromocenter configuration in CA1 pyramidal neurons of mature hippocampus [73]. Chromatin changes were shown to be accompanied by changes in epigenetic marks that are associated with open chromatin states [73]. These findings suggest that MeCP2 not only establishes chromatin structure during neurodevelopment, but also maintains the developmentally established nuclear geometry and chromatin organization in fully developed neurons during adulthood.

Chromatin organization and the architecture of the nucleus gates interactions between transcriptional regulatory sequences and promoters, regulates accessibility of gene loci to transcriptional machinery and thus influence the transcriptional dynamics in neurons [80,81]. MeCP2 KO restricted to the adult mouse brain altered the expression of several genes including *Grin2a* and *Bdnf* [70]. Furthermore, reducing MeCP2 levels in total dorsal hippocampus [73] or selectively in the DG of the hippocampus [74] in young adult mice altered the transcriptional profile of fully developed hippocampal neurons. Consistent with the repressor role for MeCP2 in transcriptional dynamics [82] and the suggested open chromatin state, a higher number of genes were upregulated than downregulated upon the reduction of MeCP2 [69,73]. Altogether, these findings indicate that disrupted chromatin structure as a result of MeCP2 depletion is accompanied by impaired transcriptomic profile in mature hippocampal neurons. Interestingly, decreasing whole-brain levels of MeCP2 was reported to reduce expressions of synaptic proteins such as CamKIIα/β, GluR2/3, GluN2A, and GABABR2 without altering their mRNA expression [24,72]. These findings suggest that in addition to the regulation at the transcriptional level, MeCP2 may regulate gene expression through post-translational mechanisms and this could explain why only subtle changes are detected upon transcriptional analysis of MeCP2-manipulated neurons.

### 3.2. Chromatin Structure and MeCP2 Regulate Activity-Dependent Gene Expression in the Adult Brain

Increasing evidence indicates that the location of genes is dynamically motile within the nucleus. Neuronal activity-induced chromosomal relocations were first reported in 1980s and corroborated in following studies [83,84,85,86]. Recently, Su and colleagues (2017) performed a comprehensive analysis of genome-wide chromatin accessibility within adult mice DG neurons and revealed how the chromatin landscape changes upon neuronal stimulation [87]. The authors demonstrated that some gene loci (such as immediate early genes (IEGs) *Arc* and *c-fos*) gained open chromatin accessibility and increased their expression, whereas fewer gene loci (such as *Gabrr1*) gained closed chromatin accessibility and reduced expression upon electroconvulsive stimulation of DG neurons [87]. More recently, Yamada and colleagues (2019) revealed that a more physiological stimulus, sensory learning, is also associated with changes in neuronal chromatin organization and *de novo* enhancer-promoter interactions in cerebellar granule neurons of adult mice brain [88]. Either through preserving an optimal chromatin state at basal conditions or through activity-dependent chromatin modifications, it is now believed that chromatin architecture has a pivotal role in the transcriptional permissiveness and response of neurons to neuronal activity. For instance, chromatin loopings were shown to regulate enhancer-promoter interactions, which correlated with the induction levels of some IEGs, such as *c-fos*, after triggering neuronal activity [89]. However, despite the central involvement of MeCP2 in chromatin maintenance of adult neurons, whether MeCP2 gates the genomic permissiveness to neuronal activity, in adulthood was not thoroughly explored. Recently, it has been shown that MeCP2 indeed preserves the transcriptional response of mature hippocampal neurons in adult mice [73]. In this study, authors lowered the levels of MeCP2 in the adult mouse hippocampus and compared the transcriptional profile of adult hippocampal neurons in basal conditions and after learning [73]. They showed that MeCP2 disruption caused a drastic impairment in the induction of activity-dependent expression of genes including *Npas4*, *Atf3*, *Dusp1*, and *Gadd45b/g* [73]. Taken together, these findings underscore the functional importance of MeCP2 in the regulation of chromatin architecture during adulthood and provides insight into how MeCP2 knockdown-related chromatin disruptions in neurons can impair the transcriptional profile of mature brain in basal conditions and in response to novel experiences.

## 4. Conclusions and Outlook

Several lines of evidence show that MeCP2 ensures proper neurodevelopment of the brain and is required to establish the chromatin structure, synaptic and functional properties of neurons. The expression of MeCP2 increases in the later stages of neurodevelopment, which coincides with the full expression of symptoms in RTT patients. Therefore, RTT has long been viewed has a disease of neuronal maturation and maintenance. In recent years it has been shown that MeCP2 also regulates the early stages of embryonic neurodevelopment, including neurogenesis and differentiation. Therefore the impairments in RTT patients and mouse models may result from cumulative deficits that start already in the early stages of development.

A few studies also demonstrate that MeCP2 has a maintenance function throughout adult life. MeCP2 preserves the developmentally established features of neuronal chromatin and modulates synaptic plasticity-related mechanisms in neurons, such as learning and memory. An emerging view is that MeCP2 fine-tunes gene expression and functions as a global chromatin organizer both during development and adulthood [48,90,91]. MeCP2, through global binding to the DNA and interaction with chromatin regulators, establishes the chromatin structure. The absence of MeCP2 may disrupt the three-dimensional chromatin architecture and lead to the described subtle changes in gene expression. However, more research needs to be carried out in order to clarify the link between the multiple functions of MeCP2 and its chromatin regulator role. It still remains to be understood whether changes in the transcriptomic profile as a result of MeCP2 dysfunction are causally linked to altered chromatin structure. Current technological advances, such as the 3C technique or ATAC sequencing, allow the investigation of chromatin structure and DNA accessibility at higher resolution. This technology should allow starting to uncover the MeCP2-dependent chromatin dynamics at gene loci resolution. It could be applied to address whether changes in gene expression as a consequence of MeCP2 depletion are associated with altered chromatin environment at the respective gene locus. Furthermore, this technique could be employed to investigate whether MeCP2 is required to maintain a permissive chromatin state for transcriptional activation at activity-regulated genes. Overall further insight into the neurobiology and function of MeCP2 would result in a deeper understanding of RTT and MDS and facilitate the design of therapeutic strategies.

## Abbreviations

MeCP2	Methyl CpG binding protein 2
RTT	Rett syndrome
MDS	MeCP2 duplication syndrome
MBD	Methyl-CpG-binding domain
ASD	Autism spectrum disorder
NID	NCoR-interacting domain
NLS	Nuclear localization signal
NTD	*N*-terminal domain
ID	Intervening domain
CTD	*C*-terminal domain
iPSC	Induced pluripotent stem cell
NPCs	Neuronal progenitor cells
GFAP	Glial fibrillary acidic protein
miRNAs	micro RNAs
KO	Knockout
E/I	Excitatory/inhibitory
CREB	cAMP response element binding protein
ATRX	Alpha-thalassemia/mental retardation syndrome X-linked protein
CTCF	CCCTC-binding factor
Bdnf	Brain-derived neurotrophic factor
DG	Dentate gyrus
NAc	Nucleus accumbens
